# Current status and future perspectives for endoscopic treatment of local complications in chronic pancreatitis

**DOI:** 10.1111/den.14926

**Published:** 2024-10-04

**Authors:** Ken Ito, Kensuke Takuma, Naoki Okano, Yuto Yamada, Michihiro Saito, Manabu Watanabe, Yoshinori Igarashi, Takahisa Matsuda

**Affiliations:** ^1^ Division of Gastroenterology and Hepatology Department of Internal Medicine Toho University Omori Medical Center Tokyo Japan; ^2^ Division of Gastroenterology and Hepatology Department of Internal Medicine Toho University Ohashi Medical Center Tokyo Japan

**Keywords:** calculi, chronic pancreatitis, endoscopy, extracorporeal shockwave lithotripsy, stricture

## Abstract

Chronic pancreatitis is a progressive disease characterized by irregular fibrosis, cellular infiltration, and parenchymal loss within the pancreas. Chronic pancreatitis treatment includes lifestyle modifications based on disease etiology, dietary adjustments appropriate for each stage and condition, drug therapy, endoscopic treatments, and surgical treatments. Although surgical treatments of symptomatic chronic pancreatitis provide good pain relief, endoscopic therapies are recommended as the first‐line treatment because they are minimally invasive. In recent years, endoscopic therapy has emerged as an alternative treatment method to surgery for managing local complications in patients with chronic pancreatitis. For pancreatic stone removal, a combination of extracorporeal shock wave lithotripsy and endoscopic extraction is used. For refractory pancreatic duct stones, intracorporeal fragmentation techniques, such as pancreatoscopy‐guided electrohydraulic lithotripsy and laser lithotripsy, offer additional options. Interventional endoscopic ultrasound has become the primary treatment modality for pancreatic pseudocysts, except in the absence of disconnected pancreatic duct syndrome. This review focuses on the current status of endoscopic therapies for common local complications of chronic pancreatitis, including updated information in the past few years.

## INTRODUCTION

In chronic pancreatitis (CP), stenosis of the main pancreatic duct (MPD) often occurs with pancreatic calculus, leading to persistent pain and other complications. The treatment options include duct decompression, resection, or a combination of both to alleviate pain, with endoscopic intervention being preferred because of its minimally invasive nature. Endoscopic treatment involves stone removal, stenting for duct strictures, and pseudocyst treatment. Here, we discuss endoscopic therapies' current state and prospects for managing CP‐associated complications.

## PANCREATIC STONES

### Extracorporeal shock wave lithotripsy

**Figure 1:**
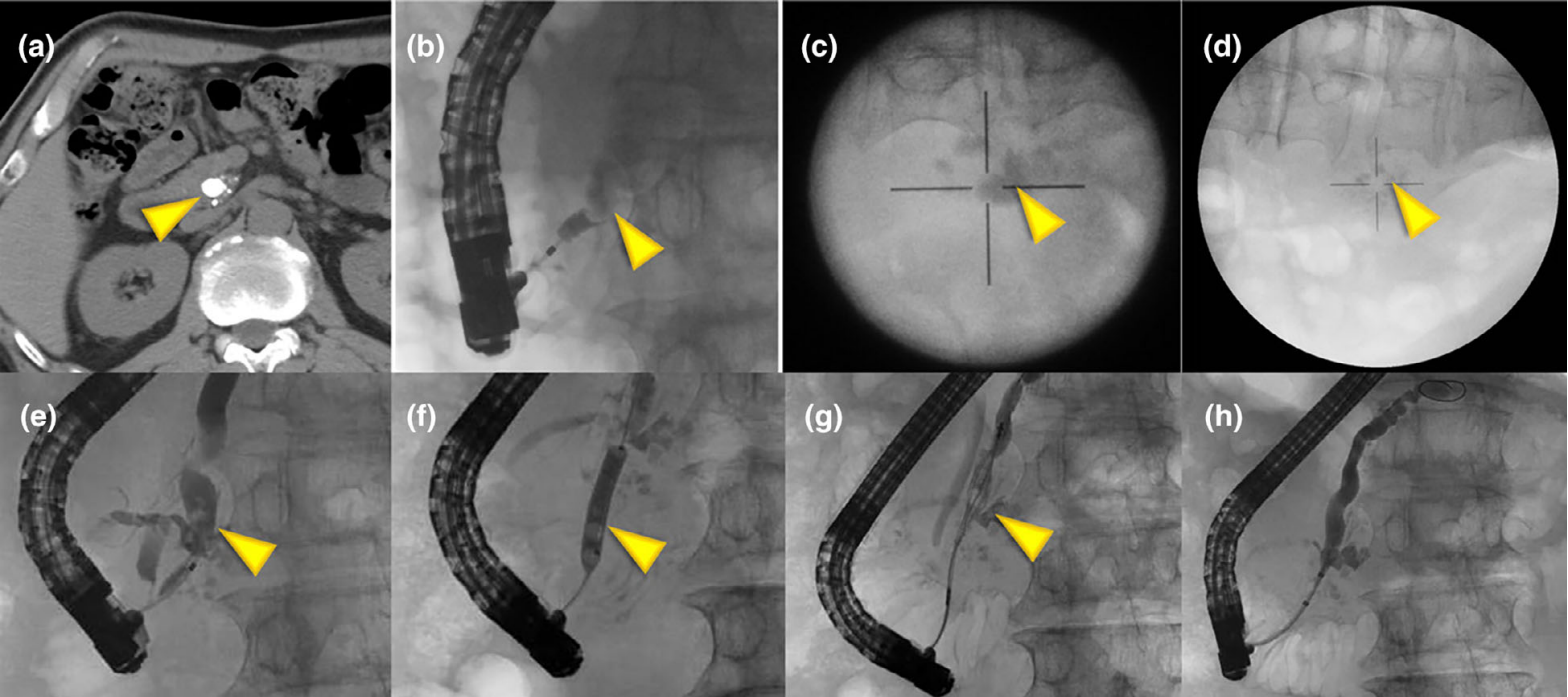
Extracorporeal shock wave lithotripsy (ESWL) and additional endoscopic stone removal. (a) Arrowhead shows symptomatic 12 mm stone at head of main pancreatic duct (MPD) (computed tomography value, 1137 Hounsfield units). (b) Endoscopic retrograde pancreatography showed impacted stone (arrowhead). (c, d) 26,000 shots of ESWL were performed and were effective (arrowhead). (e) Endoscopic retrograde cholangiopancreatography showing remaining stones in the head of MPD (arrowhead). (f) Using papillary balloon dilation. (g) Using basket forceps extraction (arrowhead), (h) Complete stone clearance.

In 1987, Sauerbruch *et al*.^1^ introduced extracorporeal shock wave lithotripsy (ESWL) for pancreatic duct stones (PDS). Electromagnetic systems are currently favored for their simplicity.^2^ Japanese studies confirmed ESWL's effectiveness, achieving stone disintegration rates of 80–100%. Consequently, ESWL became the standard nonoperative treatment for PDS.^3^, ^4^, ^5^, ^6^ Indications for ESWL include symptomatic cases with MPD dilation on imaging resulting from pancreatic head or body stone obstruction. A systematic review by van Huijgevoort *et al*.^7^ analyzed 22 studies encompassing 3868 patients with symptomatic PDS to compare ESWL with endoscopic retrograde cholangiopancreatography (ERCP) outcomes. ESWL showed promising results: 69.8% (95% confidence interval [CI] 63.8–75.5) achieved complete ductal clearance, 64.2% (95% CI 57.5–70.6) experienced pain resolution during follow‐up, and 86.3% (95% CI 76.0–94.0) achieved complete stone fragmentation. Complications such as postoperative pancreatitis and cholangitis were minimal, occurring in 4% (95% CI 2.5–5.8) and 0.5% (95% CI 0.2–0.9) of patients, respectively.^3^, ^5^, ^6^, ^8^, ^9^, ^10^, ^11^, ^12^, ^13^, ^14^, ^15^, ^16^, ^17^, ^18^, ^19^, ^20^, ^21^, ^22^, ^23^, ^24^, ^25^, ^26^ In a 2016 meta‐analysis on the efficacy and safety of ESWL, the complete ductal clearance among pooled patients was 70.69% (95% CI 68.97–72.38), similar to the findings of a 2020 meta‐analysis.^7^, ^27^ Thirteen of 27 studies in this review were published before 2000; therefore, it provides important updated information on the results of ESWL for PDS. Institutions vary in ESWL positioning, and studies yield mixed results on factors like a single stone in the pancreatic head, pancreatic stenting before ESWL, stone computed tomography (CT) value <820.5 Hounsfield units, and performing ERCP 2 days after ESWL.^9^, ^15^, ^18^, ^26^, ^28^, ^29^, ^30^, ^31^ Recent studies suggest that differences between ESWL lithotripters may affect their efficacies in lithotripsy types, necessitating further investigations.^2^, ^6^, ^32^ ESWL's specific limits for pancreatic stones remain undefined. Studies on ESWL shot details reveal complete stone clearance rates ranging from 32% to 100% with shot counts between 1500 and 18,000.^8^, ^9^, ^12^, ^15^, ^18^, ^23^, ^33^, ^34^, ^35^, ^36^, ^37^ Additionally, reports with more than 20,000 shots showed complete stone clearance of 44–93.7%.^26^, ^32^ Table 1 summarizes the ESWL shot schemes in the literature.^8^, ^9^, ^12^, ^15^, ^18^, ^19^, ^26^, ^32^, ^34^, ^35^, ^36^, ^37^, ^38^, ^39^, ^40^ In cases in which ESWL monotherapy fails to remove pancreatic stones, additional endoscopic therapies such as basket forceps lithotripsy and endoscopic pancreatic stenting (EPS) offer high success rates, with complete stone disappearance rate reaching 76–100%, indicating a good outcome (Fig. 1).^32^, ^41^ However, in a randomized controlled trial (RCT), combination therapy of endoscopic treatment and ESWL was shown to increase patient care costs without improvements in pain relief outcomes.^39^ Therefore, use of the combination therapy should be restricted to patients with MPD strictures or cases where spontaneous stone clearance is not achieved after adequate fragmentation with ESWL. Stone removal during stent placement is challenging. Patients with PDS often have MPD strictures as a complication, requiring stenting. Takuma *et al*.^42^ reported morphological changes in 31.1% of the 132 patients who underwent 10F S‐type EPS implantation during ESWL treatment. Hence, the selection of stent placement for MPD stricture during ESWL treatment for PDS should be carefully considered.^42^ The current management of asymptomatic PDS remains controversial. International consensus guidelines (International Association of Pancreatology, the American Pancreatic Association, the Japan Pancreas Society, and the European Pancreatic Club) suggest that painless PDS is not recommended for endoscopic or surgical treatment to improve endocrine/exocrine function or prevent pancreatic cancer.^43^ However, the Japanese Clinical Guidelines for Endoscopic Treatment of Pancreatolithiasis suggest treating asymptomatic PDS without parenchymal atrophy to potentially improve pancreatic functions.^41^ These discrepancies underscore the need for future multicenter studies.

**Table 1:** Previous studies with detailed outcomes on the number of sessions and/or total number of shots for third‐generation extracorporeal shock wave lithotripsy (ESWL) lithotripters

Author, year	ESWL device		n	Stone single/diffuse	Stone location (head)	Stone size	MPD stricture (%)	Complete stone (%)	ESWL sessions	ESWL total shots
Delhaye *et al*., 1992	Lithostar	Siemens, Erlangen, Germany	123	47/76	107^†^	13 mm	59	72 (59)	1.8 (1–11)	5142
Costamagna *et al*., 1997	Lithostar plus	Siemens, Erlangen, Germany	35	11/24	34	NA	NA	30 (74.3)	1.9 (1–4)	8417 (2900–18,000)
Brand *et al*., 2000	Lithostar prototype	Siemens, Erlangen, Germany	48	5/43	26	13 mm	NA	21 (44)	13 (2–74)	22,100 (1700–150,900)
Kozarek *et al*., 2002	Dornier HM3	Dornier Medtech, Munich, Germany	40	11/29	NA	13 mm	19	40 (100)	1, 35 2, 4 3, 1	2344 (1800–2400)
Conigliaro *et al*., 2006	Modulith, SL20, SLX	Storz Medical AG, Switzerland	4	NA	NA	NA	NA	3 (75)	1 (1–3)	2100 (1500–2000)
Dumonaceau *et al*., 2007	Lithostar plus	Siemens, Erlangen, Germany	55	NA	43	ESWL alone 10.5 mm combined 8 mm	NA	NA	ESWL monotherapy 2 (1–3) combined 2 (1–4)	NA
Merrill *et al*., 2011	HM3/Modulith SLX‐F2	Dornier Medtech, Munich, Germany/Storz Medical AG Switzerland	27	11/16	23	<5 mm, 8 5–10 mm, 4 10–20 mm,e 15	NA	Underwent ERCP more than 2 days after ESWL 9 (82) <2 days Post‐ESWL 3 (16)	NA	3000–5000
Milovic *et al*., 2011	Minilith SL 1	Storz Medical AG, Switzerland	32	10/22	32	NA	NA	13 (40.6)	4 (2.75–8.5)	6800 (4225–15,425)
Ohyama *et al*., 2015	Modulith SLX	Storz Medical AG, Switzerland	128	55/73	98	11.1 mm	57	66 (51.6)	5.2	5000
Hu *et al*., 2016	Compact Delta II	Dornier Med Tech, Wessling, Germany	214	44/170	175	NA	NA	155 (72.4)	2.2 (1–8)	10,419 ± 8648
Li *et al*., 2016	Compact Delta II	Dornier Med Tech, Wessling, Germany	59	5/54^‡^	14	5–10 mm, 22 10–20 mm, 29 20–30 mm. 6 ≥30 mm, 2	NA	ESWL single 6 (10.2) combined with ERCP 38 (67.2)	2	9920.34 ± 5436.84
Wang *et al*., 2018	Delta Compact	Dornier Med Tech, Wessling, Germany	50	NA	44	NA	NA	37 (77.1)	1.0 (1–6)	8432 (6484.4)
Tandan *et al*., 2019	Delta Compact	Dornier Med Tech, Wessling, Germany	5124	3851/1273	2824	NA	NA	3722 (72.6)	2.2 (1–8)	NA
Hao *et al*., 2019	Delta Compact	Dornier Med Tech, Wessling, Germany	72	10/62	69	5–10 mm, 32 10–20 mm, 26 20–<30 mm, 10 ≥30 cm, 4	NA	53 (73.6)	2.0 (1–5)	9812 ± 5000
Ito K *et al*., 2022	Modulith SLX‐F2/Lithostar, Lithoskop	Storz Medical AG Switzerland/Siemens, Erlangen, Germany	208	55/103	32/126	9.7 mm ± 3.2/10.4 mm ± 4.2	43.5/88	30 (93.7)/87 (69.0)	10.4 (1–145)	20,990 (1500–217,500)

^†^ Head alone, and including head and at least another location.

^‡^ 4<5 patients, 4≦54 patients.

ERCP, endoscopic retrograde cholangiopancreatography; MPD, main pancreatic duct; NA, not available.

### Intracorporeal fragmentation using peroral pancreatoscopy (POP)

**Figure 2:**
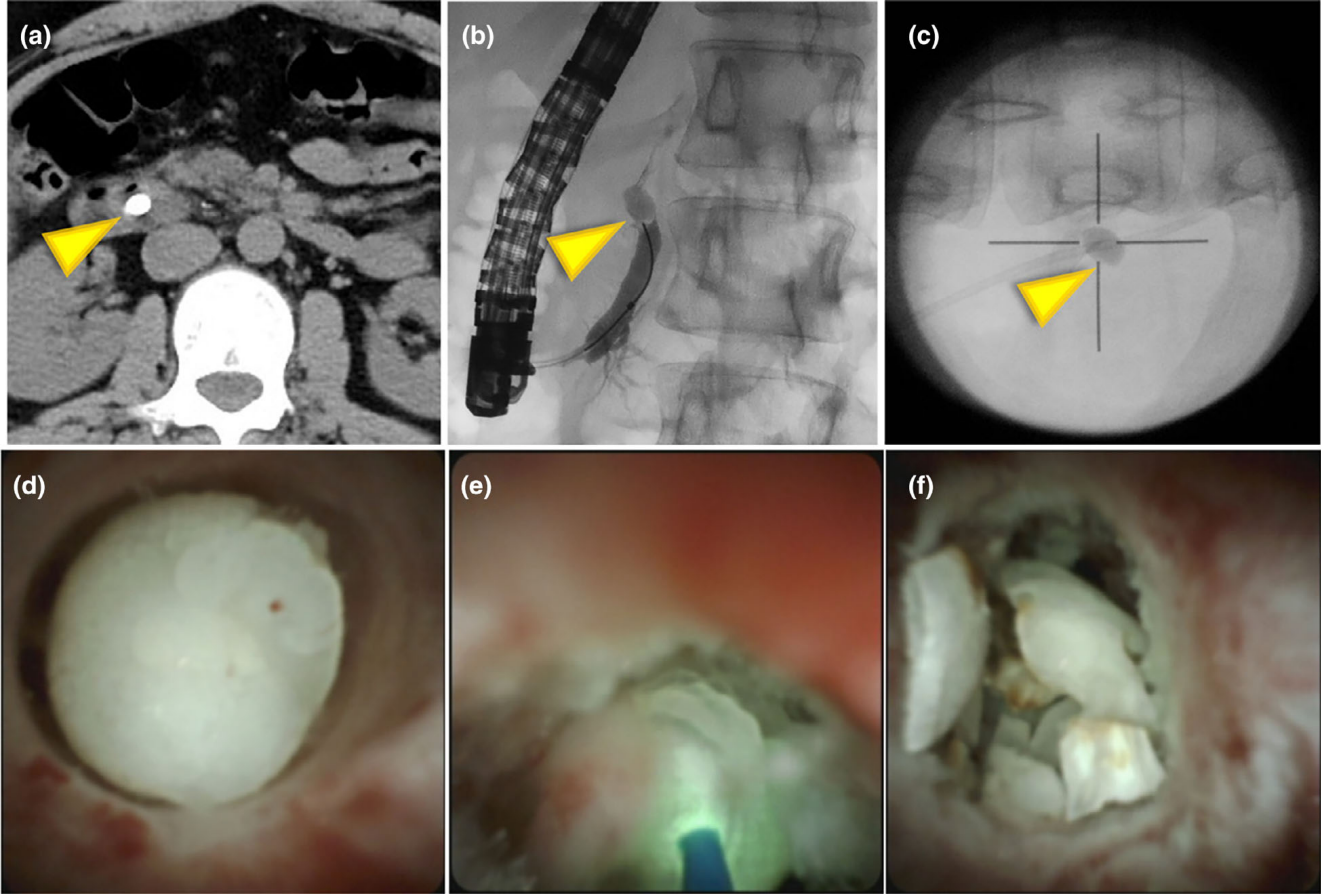
A case of refractory stone fragmented using laser lithotripsy. (a) Symptomatic 10 mm stone at the head of the main pancreatic duct (computed tomography value: 1750 Hounsfield units). (b) Endoscopic retrograde pancreatography showed impacted stone. (c) Five sessions of extracorporeal shock wave lithotripsy were performed; the procedures were not effective. (d) Use of peroral pancreatoscopy (POP) (SpyGlass DS; Boston Scientific). (e) Performing laser lithotripsy. (f) POP showing stone fragmentation.

Intracorporeal lithotripsy, using techniques like electrohydraulic lithotripsy (EHL) and laser lithotripsy (LL), has gained interest as an alternative therapy. Recent sporadic reports of systematic reviews and meta‐analyses have focused on POP‐guided lithotripsy.^44^, ^45^, ^46^, ^47^, ^48^ The introduction of innovative devices like the single‐operator cholangiopancreatoscopy system (SpyGlass DVS; Boston Scientific, Natick, MA, USA) in 2007 and its digital version (SpyGlass DS; Boston Scientific) in 2015 has expanded therapeutic options for PDS (Fig. 2). In a comprehensive analysis of 15 studies covering 218 cases of POP‐guided EHL and 155 cases of LL, technical success rates ranged from 37.5% to 100%.^16^, ^49^, ^50^, ^51^, ^52^, ^53^, ^54^, ^55^, ^56^, ^57^, ^58^, ^59^, ^60^, ^61^, ^62^ Among 10 studies using EHL alone, the pooled technical and clinical success rates were 90.9% (95% CI 88.3–95.7) and 89.8% (95% CI 87.2–95.2), respectively.^16^, ^47^, ^49^, ^51^ Some studies reported lower success rates attributed to difficulties in maneuvering POP around severe MPD strictures, unstable POP positioning near the papilla, and using POP as a rescue therapy after failed ESWL attempts.^16^, ^51^ Among 10 studies using LL alone, the technical and clinical success rates were 88.4% (95% CI 85.9–95.1) and 85.8% (95% CI 80.6–91.6), respectively.^47^, ^50^, ^52^, ^53^, ^54^, ^55^, ^59^, ^60^, ^61^, ^62^ Since 2015, studies have shown the effectiveness of POP‐guided lithotripsy with EHL/LL for pancreatic duct stones, achieving 80–100% effectiveness rates. Despite different mechanisms (EHL producing high‐amplitude hydraulic pressure waves and LL generating mechanical shockwaves through repeated laser energy pulses applied to stones),^63^, ^64^ no statistically significant difference in technical success between EHL and LL was observed, with success rates of 90.9% and 88.4%, respectively.^47^ Adverse events, including pancreatitis, abdominal pain, hemorrhage, MPD perforation, contrast extravasation, and bacteremia, were reported in 12.1% (95% CI 8.7–15.5) of cases.^16^, ^49^, ^50^, ^54^, ^55^, ^56^, ^57^, ^58^, ^59^, ^60^, ^61^, ^62^ The outcomes of POP‐guided lithotripsy are promising, with single‐session stone fragmentation expected compared to ESWL, which often requires multiple sessions. However, these studies had several limitations. First, most of them were conducted in tertiary care referral centers, potentially not reflecting the outcomes seen with less experienced operators. Additionally, our analysis included retrospective studies, which contributed to a selection bias. While the European Society of Gastrointestinal Endoscopy (ESGE) guidelines encourage considering POP‐guided lithotripsy for ESWL‐resistant stones, only four studies reported pre‐ESWL attempts.^16^, ^49^, ^59^, ^61^ Additionally, there is scarce research comparing POP‐guided lithotripsy techniques such as LL or EHL with ESWL. An ongoing multicenter RCT in the United States compares POP‐guided therapy with ESWL. This study aims to determine the most effective lithotripsy method for refractory PDS removal and assess its effects on the quality of life and pain in chronic calcific pancreatitis patients, with promising anticipated results (ClinicalTrials.gov identifier: NCT04115826).^65^ LL offers advantages over EHL: (i) higher probe durability; (ii) superior cost‐effectiveness, reducing per‐case running costs; (iii) safe for patients with pacemakers (EHL is contraindicated); and (iv) potentially less invasive to the luminal wall (LL directs energy more precisely compared to EHL's radial spread). Although anecdotal experience and clinical practice patterns may suggest that LL is preferable to EHL in POP, there was no statistical difference between EHL and LL; however, the success rate of EHL had an increasing trend compared with that of LL. Further large‐scale RCTs are needed to compare EHL‐POP and LL‐POP with ESWL and determine whether POP could potentially replace ESWL as the first‐line treatment for pancreatolithiasis.

## PANCREATIC DUCT (PD) STRICTURES

Chronic pancreatitis frequently involves MPD strictures, causing persistent pain because of increased intraductal pressure from stagnant pancreatic fluid. EPS offers effective MPD decompression and pain relief. Studies on EPS indicate high insertion success rates (85–98%), rapid pain relief in most patients (65–98%), and long‐term pain relief in a significant portion (32–68%), highlighting its utility and safety in treating pancreatic diseases (Table 2).^66^, ^67^, ^68^, ^69^, ^70^, ^71^, ^72^, ^73^, ^74^, ^75^ Various approaches have been considered for the timing, type, and duration of EPS placement. First, studies have indicated that stent placement is typically performed after stone fragmentation and removal.^10^, ^30^, ^76^ The average duration of stent insertion was 10.6 (range 3.2–23) months across 18 series involving 811 patients.^67^, ^68^, ^69^, ^71^, ^72^, ^73^, ^74^, ^75^, ^77^, ^78^, ^79^, ^80^, ^81^, ^82^, ^83^, ^84^, ^85^ Several designs have been proposed regarding the stent shape, including straight, S‐shaped, winged, and stents with or without sideholes.^86^, ^87^ In prospective studies, stents with large sideholes have been suggested to occlude less frequently than other types. However, this finding is based on few patients with CP.^88^ Regarding stent diameter, in a retrospective study involving 163 patients with CP, patients treated with stents ≤8.5F were 3.2 times more likely to be hospitalized for abdominal pain than those treated with 10F stents.^89^ Finally, regarding stent exchange timing, in on‐demand stent exchange policy, pancreatic sepsis was observed in 5.2% (15/288) of the patients according to four studies, with two patients requiring surgery for pancreatic abscesses.^66^, ^67^, ^70^, ^80^ Conversely, in 12 studies in which routine stent exchange was performed at shorter intervals (3 months), no septic complications were reported in 521 patients.^68^, ^69^, ^71^, ^72^, ^73^, ^75^, ^77^, ^78^, ^81^, ^82^, ^84^, ^90^ Based on these data, it is recommended to replace a single 10F plastic stent (PS) for painful dominant MPD strictures at least every 6 months and to keep it in place for 1 year. During this treatment, stenosis resolution was achieved in 9–50% cases, with a long‐term clinical success rate of 32–94%.^66^, ^67^, ^68^, ^71^, ^91^, ^92^, ^93^ Refractory MPD strictures are symptomatic dominant strictures that persist or recur after 1 year of single pancreatic stent placement. Insertion of multiple side‐by‐side PS is effective in managing refractory strictures; however, available data are still limited. In a study including 48 patients, multiple side‐by‐side PS were used to treat refractory MPD strictures that were to improve with a single PS after implanting an average of three (diameter, 7–11.5F; length, 3–7 cm) PS through the main or minor papilla for 6–12 months. The results of the study showed that 74.4% of patients remained asymptomatic and 25.6% experienced pancreatitis recurrence or pancreatic‐type pain after a mean follow‐up of 9.5 years (0.3–15.5 years) after stent removal, suggesting that this method may be more beneficial than conventional treatment using a single PS.^36^, ^94^ Recent reports have highlighted using fully covered self‐expandable metal stents (FCSEMS) in cases of stubborn PD strictures.^93^, ^94^, ^95^, ^96^, ^97^, ^98^, ^99^, ^100^, ^101^, ^102^, ^103^, ^104^ In a systematic review and meta‐analysis comparing FCSEMS and multiple side‐by‐side PS for treating MPD strictures, no significant differences were found in pain improvement (88% vs. 89%) or stricture recurrence (8% vs. 11%) among 192 and 106 patients, respectively.^105^ However, patients treated with FCSEMS required fewer ERCPs, though they experienced a significantly higher adverse event rate than those with multiple PS (39% vs. 14%). Adverse events related to self‐expandable metal stents (SEMS) in the PD include stent migration, bile duct obstruction, challenges in removal, and formation of de novo MPD strictures.

**Table 2:** Placement of single and multiple plastic stents for main pancreatic duct strictures in patients with chronic pancreatitis

Author, year	*n*	Stent diameter (F)	Number of stents	Duration of stent placement (months)	Long‐term follow‐up (months)	Pain relief, immmediate (%)	Pain relief, long‐term (%)	Complications (%)	Recurrent pain (%)	Transition to surgery (%)
Cremer, 1991	75	10	Single	12.0	37.0	94	NA	Stent clogging 10.6	NA	15.0
Ponchon, 1995	23	10	Single	6.0	14.0	74	52	Pancreatitis 39.0	60.8	13.0
Smits, 1995	49	10	Single	6.0	34.0	82	82	Early 18.0, stent‐related 55.0	31.2	8.1
Binmoeller, 1995	93	5‐7‐10	Single	15.7	58.0	74	65	Pancreatitis 4.3, stent clogging 2.1	13.9	25.8
Morgan, 2003	25	5–7–8.5	Single	NA	NA	65	NA	NA	NA	NA
Vitale, 2004	89	5–7–8.5	Single	6.0	43.0	83	68	19.0	26.9	12.3
Gabbrelli, 2005	22	8.5–11.5	Single	NA	66.0	100	55	NA	22.7	18.1
Bartolli, 2005	39	7–9	Single	6.0	15.7	100	78	30.6	45.4	10.2
Eleftheriadis, 2005	100	8.5–10	Single	6.0	69.0	70	62	Sepsis 11.0, pancreatitis 11.0, impaction of stent 2.0, migration of stent 2.0	30.0	4.0
Farrnbacher, 2006	98	NA	Single	0.5	36.0	NA	67	NA	24.0	14.0
Costamagna, 2006	19	8.5–11.5	Multiple	≥3.0	38.0	84	NA	Proximal migration 3.0	10.5	0.0
Ishihara, 2006	20	10	Single	12.0	21.0	95	90	NA	10.0	0.0
Weber, 2007	19	7–8.5–10–11.5	Single	5.6	24.0	89	83	Stent occlusion or dislocation 31.5	17.6	24.0
Cahen, 2007	19	10	Single	7.0	24.0	NA	32	58.0	36.8	21.0
Boursier, 2008	13	8.5–10	Single	4.5 ± 3.0	11.0	85	92	Pancreatitis 10.0	NA	0.0
Ito, 2018	59	10	Single	9.0	27.0	90.2	NA	3.6	41.5	6.7
Tringali, 2019	48	7–11.5	Single	6.0–12.0	9.5	NA	74.4	Pancreatitis 4.2	25.6	0.0

NA, not available.

In 2022, a multicenter RCT included 67 patients with painful PD strictures; a novel option for PD stricture, FCSEMS, was utilized.^106^ The technical success rate was 97% (65/67); however, stent migration occurred in 47.7% (31/65), with 50.7% requiring PS placement within 90 days after the insertion of FCSEMS. Furthermore, a 2023 retrospective study found that after FCSEMS placement in 35 cases of CP‐related refractory stricture, a 48.6% (17/35) stent‐induced de novo stricture was reported during a mean follow‐up period of 136 (range 85.8–145.5) months.^107^ As a future outlook, high‐quality, well‐designed FCSEMS are expected to be developed and provide evidence‐based data on their short‐ and long‐term efficacy, safety, and optimal duration of placement.

**Figure 3:**
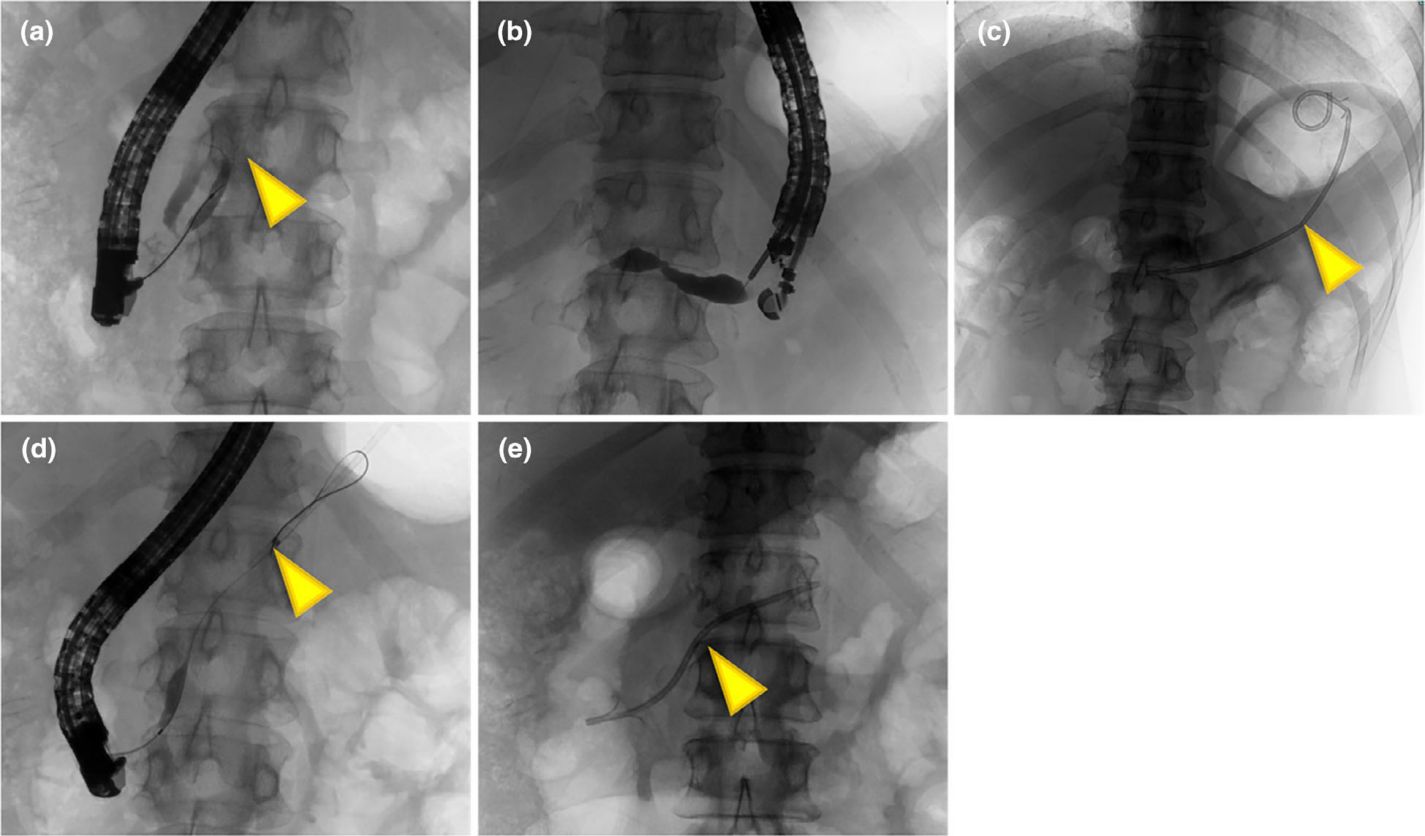
Endoscopic ultrasound‐guided pancreatic duct drainage. (a) Endoscopic retrograde pancreatography showing severe stricture (arrowhead). (b) A 19G needle was punctured into the main pancreatic duct (MPD), and a pancreatogram shows the dilated MPD. (c) Pancreaticogastrostomy was completed using a 7F plastic stent (PS) (arrowhead). (d) After 6 month, stricture (arrowhead) was possible to pass though by transpapillary approach. (e) 10F PS placement (arrowhead).

Interventional endoscopic ultrasound‐guided pancreatic duct drainage (EUS‐PD) is recommended for patients with symptomatic MPD obstruction after failed conventional endotherapy or surgically altered anatomy (Fig. 3). The procedure includes puncturing the MPD via the gastric or duodenal wall, inserting a guidewire, and performing transpapillary (rendezvous technique [RV]) or transmural drainage. Studies, both small‐scale single‐center and larger multicenter, document these procedures involving 36–80 patients.^108^, ^109^, ^110^, ^111^, ^112^, ^113^, ^114^, ^115^, ^116^, ^117^, ^118^, ^119^, ^120^, ^121^, ^122^, ^123^, ^124^, ^125^ Table 3 summarizes previous reports of EUS‐PD following endoscopic retrograde pancreatography (ERP) failure, including cases involving RV methods.^110^, ^111^, ^112^, ^121^, ^123^, ^126^, ^127^, ^128^, ^129^ Immediate pain relief after successful EUS‐guided MPD access and drainage is common in obstructive CP (50–100%), with substantial relief in 70–90% of patients. However, sustained relief declines over time.^122^, ^123^ Pancreatic stiffness in certain CP cases can complicate puncturing and dilation, emphasizing the need for safety precautions. Unsuccessful EUS‐guided access occurs in about 10% of cases, with moderate‐to‐severe complications of around 10% in larger series, including severe pancreatitis, perforation, bleeding, and hematoma.^108^, ^109^, ^110^, ^111^, ^112^, ^122^, ^123^, ^124^ Therefore, these techniques are recommended to be performed in tertiary centers.

**Table 3:** Previous studies on endoscopic ultrasound‐guided pancreatic duct drainage (EUS‐PD) following endoscopic retrograde pancreatography (ERP) failure

Author, year	*n*	Indication for EUS‐PD	Procedure	Technical success, %	Clinical success, %	Adverse events	Details of adverse events
Francois *et al*., 2002	4	Failed ERP	RV	100.0	75.0	25.0	Stent dislocation
Mallery *et al*., 2004	4	Failed ERP, SAA	RV	25.0	25.0	25.0	Fever
Kahaleh *et al*., 2007	13	Failed ERP, SAA	TD	76.9	100.0	15.4	Bleeding, perforation
Tessier *et al*., 2007	36	Failed ERP, SAA	TD	91.7	75.8	13.9	Pancreatitis, hematoma
Will *et al*., 2007	12	Failed ERP, SAA	RV, TD	69.2	NA	42.9	Abdominal pain, bleeding, perforation
Brauer *et al*., 2009	8	Failed ERP	RV, TD	87.5	37.5	0.0	‐
Barkay *et al*., 2010	12	Failed ERP	RV	33.3	NA	9.5	Peripancreatic abscess, pancreatitis
Ergun *et al*., 2011	20	Failed ERP, SAA	RV, TD	90.0	65.0	10.0	Bleeding, fluid collection
Shah *et al*., 2012	25	Failed ERP, SAA	RV, TD	54.5	NA	16.0	Pancreatitis, pneumoperitoneum
Vila *et al*., 2012	19	EUS‐guided PD drain	RV, TD	57.9	63.2	26.3	‐
Fujii *et al*., 2013	43	Failed ERP, SAA	TD	74.4	93.1	37.2	Abdominal pain, abscess, pancreatitis
Kurihara *et al*., 2013	17	Failed ERP, SAA	RV, TD	88.2	88.2	5.9	Aneurysm
Will *et al*., 2015	94	Failed ERP, SAA	RV, TD	56.6	28.9	21.6	Bleeding, pancreatitis, abscess, fluid collection, perforation, retention cyst, aspiration, ulcers
Oh *et al*., 2016	25	Failed ERP, SAA	TD	100.0	100.0	20.0	Abdominal pain, bleeding
Tyberg *et al*., 2017	80	Failed ERP, SAA	RV, TD	88.8	91.5	20.0	Pancreatitis, pancreatic fluid collection, abdominal pain, bleeding, MPD leak, perforation
James *et al*., 2018	5	Failed ERP	TD	100.0	100.0	0.0	‐
Matsunami *et al*., 2018	30	Failed ERP, SAA	TD	100.0	100.0	23.3	Abdominal pain, abscess, ulcer
Uchida *et al*., 2018	15	Failed ERP, SAA	TD	86.7	92.3	26.7	Peritonitis, stent dislocation, bleeding
Dalal *et al*., 2020	44	Failed ERP, SAA	RV, TD	88.6	81.8	22.7	Abdominal pain, pancreatitis, fever, bleeding
Suzuki *et al*., 2022	20	Failed ERP, SAA	RV, TD	95.0	100.0	20.0	Pancreatic juice leakage, pancreatitis

NA, not available; RV, rendezvous; SAA, surgically altered anatomy; TD, transmural drainage.

## OPTIMAL TIME FOR SURGERY

Many patients with CP present complex features (combination of strictures and stones), making it challenging to generalize ductal obstruction in these patients. Early intervention in patients with a single obstruction or stone may treat the condition and lead to long‐term pain relief after endoscopic pancreatic duct drainage, with disease duration as the only parameter.^22^ The main concern is the delayed timing of surgical treatment because of excessive reliance on endoscopic therapy and ESWL. For patients experiencing persistent pain after endoscopic therapy, appropriate timing for surgery is crucial to prevent disease progression and provide better pain management. A systematic review and meta‐analysis of three RCTs and two retrospective studies including 602 patients showed no significant differences in postoperative complication rates (odds ratio [OR] 0.91, 95% CI 0.51–1.61, *P* = 0.74, *I* ^2^ = 38.8%), endocrine dysfunction resulting from CP (OR 1.18, 95% CI 0.63–2.20, *P* = 0.61, *I* ^2^ = 28.24%), or exocrine dysfunction (OR 1.78, 95% CI 0.66–4.79, *P* = 0.25, *I* ^2^ = 30.97%); however, early surgery demonstrated higher pain relief compared to endoscopic interventions (OR 0.46, 95% CI 0.27–0.80, *P* = 0.01, *I* ^2^ = 17.65%).^74^, ^77^, ^130^, ^131^, ^132^, ^133^ In an RCT involving 39 cases of symptomatic CP and distal obstruction of the MPD without an inflammatory mass, use of the mean Izbicki pain score allowed for adequate pain evaluation, confirming that surgical drainage via pancreaticojejunostomy leads to effective decompression.^74^ In an RCT comprising 88 cases of painful CP, pancreatic resections such as Frey, Smith, and Beger procedures were incorporated alongside surgical drainage in cases with inflammatory masses in the pancreatic head. Pain was evaluated using the Izbicki score, demonstrating the superiority of surgical intervention.^132^, ^134^, ^135^ Although it may be rational to initially treat CP‐associated pain with endoscopic therapy, endoscopic drainage is considered less effective than surgery in patients with symptoms associated with complex pathologies. Most patients and their referring physicians tend to prefer noninvasive procedures during the initial stage of treatment; therefore, generalizing the early surgery strategy may still be difficult despite these results.

## PSEUDOCYST MANAGEMENT

Pancreatic pseudocysts are formed when pancreatic ducts rupture because of pancreatitis or trauma, leading to accumulation of pancreatic fluid collection (PFC) and necrotic material. They are among the most common complications observed during acute pancreatitis and CP. Acute pseudocysts are defined in the revised Atlanta classification as acute peripancreatic fluid collection persisting beyond 4 weeks. Chronic pseudocysts, on the other hand, are associated with CP, characterized by a definite wall and the absence of preceding acute pancreatitis episodes.^136^, ^137^ PFC occurs in approximately one third of patients. Spontaneous resolution of PFC is rare (0–27%), mainly in small (<4 cm) and localized cases, and asymptomatic cases can be monitored.^138^ Drainage is recommended for symptomatic pseudocysts with symptoms like abdominal pain, fever, or upper digestive tract obstruction. Additionally, drainage is warranted for pseudocysts causing vascular or biliary compression, pancreato‐pleural fistula, those >5 cm in size with no reduction after 6 weeks, and those with significant duct distortion.^139^ However, in clinical practice, there are often situations where a wait‐and‐see approach for endoscopic cyst drainage until that time is not feasible. Therefore, early intervention is recommended in cases where CP‐related pseudocysts exhibit symptoms (abdominal pain, gastric outlet obstruction, early satiety, weight loss, or jaundice) or complications (infection, bleeding, rupture, or adjacent hollow strictures) within 6 weeks.^140^, ^141^ Endoscopic drainage is preferred for uncomplicated CP pseudocysts accessible via endoscopy rather than percutaneous or surgical methods. In a meta‐analysis (five articles, 255 patients), no significant differences were observed in treatment‐related complication rates (OR 1.63, 95% CI 0.71–3.73), pseudocyst recurrence rates (OR 1.53, 95% CI 0.37–6.39), hospital stay, or cost between endoscopic and surgical treatments.^142^ A systematic review of four studies (229 patients) showed a higher technical success rate for EUS‐guided transmural drainage than conventional methods (risk ratio [RR] 12.38; 95% CI 1.39–11.22).^143^ However, endoscopic transpapillary drainage is recommended for small (<5 cm) pseudocysts communicating with the main pancreatic duct in the pancreatic head or body.^144^, ^145^

For pseudocysts containing only liquid, double‐pigtail PS are typically favored over lumen‐apposing metal stents (LAMS) because of cost considerations. Removing transmural PS at least 6 weeks after pseudocyst regression if MPD disruption is ruled out and suggests considering long‐term placement of transmural double‐pigtail PS in disconnected PD syndrome cases.^146^, ^147^ For cases without disconnected PD syndrome and an expected indwelling duration of less than 6 weeks, FCSEMS such as LAMS may be considered. A meta‐analysis of six retrospective studies (504 patients) compared LAMS with multiple PS for PFC treatment, revealing higher clinical success rates (RR 2.70, 95% CI 1.49–5.00) and lower morbidity with LAMS (RR 0.39, 95% CI 0.18–0.84). However, PFC was only present in 11% of patients, and a cost‐effectiveness analysis favored multiple PS.^124^ Based on this information, the most appropriate treatment for each individual case must be considered, based on factors such as cost‐effectiveness of treatment, pathophysiology, duration of placement, treatment success rate, and complication rate in pseudocyst therapy.

## BILIARY STRICTURES

Biliary strictures are a common complication of CP, occurring in 10–30% of cases.^91^, ^148^ Although some nondilated strictures may not require treatment, those causing cholangitis and obstructive jaundice should be addressed. Untreated CP‐related biliary strictures can lead to secondary biliary cirrhosis in approximately 7% of cases.^38^, ^149^ Endoscopic stenting is recommended for CP patients with prolonged biliary obstruction (lasting over 4 weeks) marked by jaundice or elevated serum alkaline phosphatase and/or bilirubin levels. If a single PS is ineffective for benign biliary stricture, multiple plastic stenting (MPS) or FCSEMS is an alternative for endoscopic biliary drainage. Three RCTs have compared MPS with FCSEMS for benign biliary strictures. A 2015 RCT in Finland involving 60 cases of CP compared the safety and feasibility of FCSEMS and MPS. The study found that a 6‐month treatment with either six 10F PS (three 10F PS at randomization, plus three more at 3 months, making a total of six 10F PS) or with one 10 mm FCSEMS resulted in good long‐term relief of biliary stricture caused by CP,^150^, ^151^, ^152^ with a 2‐year stricture‐free success rate of 90% (95% CI 72–97%) in the PS group and 92% (95% CI 70–98%) in the FCSEMS group.^153^ ESGE and American Society of Gastrointestinal Endoscopy (ASGE) guidelines emphasized the need for additional long‐term RCT data on the endoscopic management of pancreatic diseases. Accordingly, an international multicenter study comparing the 2‐year efficacy and safety outcomes of MPS and FCSEMS for CP with benign biliary strictures was conducted in 2021.^154^ The PS group underwent three to four side‐by‐side MPS stenting procedures with at least two 8.5 or 10F PS during the 12‐month treatment period. FCSEMS involved the placement of a single 8 or 10 mm stent for 12 months, with the choice of stent size left to the investigator's discretion. Stricture resolution status at 24 months showed similar efficacy and safety for 12‐month treatment using MPS and FCSEMS (77.1% vs 75.8%; *P* = 0.008 for noninferiority intention‐to‐treat analysis), though requiring fewer ERCPs over 2 years. In a 2016 US RCT, FCSEMS (92.6%) was not inferior to MPS (85.4%) in stricture resolution at 12 months. However, the inclusion criteria covered benign biliary strictures from CP and posttransplant strictures, resulting in insufficient power for subgroup analysis comparing FCSEMS and MPS efficacy in each entity.^155^ Given this background, the number of RCTs needs to be increased, and further large‐scale, long‐term studies are required to clarify the optimal indications and management methods for FCSEMS and MPS. Therefore, the first choice for treating benign biliary strictures associated with CP is FCSEMS or MPS, but it is also necessary to address the limitations of treatment indications. Although surgical outcomes are from the 2000s, studies report promising success rates ranging from 73% to 90%.^156^, ^157^, ^158^ Therefore, the appropriate treatment duration for biliary stenting is 2 years. In cases in which the treatment is ineffective or where treatment compliance is low, making regular ERCP difficult, it is necessary to consider surgical options (such as choledochojejunostomy or choledochoduodenostomy) instead of persisting with endoscopic treatment.^148^

## CELIAC PLEXUS BLOCK AND NEUROLYSIS

Pain management is a major challenge for patients with CP. Concerns about dependence and abuse of oral analgesics have led to the use of EUS‐guided celiac plexus neurolysis (EUS‐CPN) and EUS‐guided celiac plexus block (EUS‐CPB). CPN is a treatment method aimed at achieving a more lasting effect through long‐term transmission blockade using chemical ablation, such as alcohol or phenol.^159^ In CPN, it is believed that alcohol or phenol injection provides a lasting effect.^160^, ^161^ However, in a prospective study involving 90 CP patients with abdominal pain, the overall pain relief effect of CPB was temporary, with pain improvement in 55%, and only 10% experiencing improvement lasting beyond 24 weeks.^162^

Celiac plexus block involves a combination of local anesthetics and steroids, with diffusion injection commonly performed over the superior mesenteric vein. However, its duration of effect is approximately 2–3 months, and repeat injections for those unresponsive to initial treatment are of uncertain efficacy.^162^, ^163^ Adverse effects, including transient worsening of pain, diarrhea, and hypotension, occur in about 40% of cases.^95^, ^164^, ^165^, ^166^, ^167^, ^168^ Therefore, this procedure should be reserved for severe CP cases unresponsive to other therapies.

Endoscopic ultrasound‐guided celiac ganglion neurolysis (EUS‐CGN) has recently been used for CP and pancreatic adenocarcinoma. However, a recent randomized multicenter trial including patients with painful upper abdominal cancer suggests that the long‐term prognosis of EUS‐CGN is poor and may shorten survival, therefore resulting in limited use.^169^

## ENDOSCOPIC TREATMENTS FOR PSEUDOANEURYSM

Chronic pancreatitis can result in vascular complications such as venous thrombosis, varices, and pseudoaneurysm bleeding. About 10% of cases involve pseudoaneurysms, with rupture rates between 2% and 10%. Although intermittent abdominal pain with gastrointestinal bleeding is common, severe bleeding may lead to hemorrhagic shock, necessitating immediate intervention.^170^, ^171^ Digital subtraction angiography (DSA)–guided endovascular intervention with coils or hemostatic agents is preferred due to its high technical (89–99%) and clinical success rates (74–88%), making it the primary treatment option.^172^, ^173^ However, technical challenges may arise, such as when the pseudoaneurysm has a short neck or when the vascular area is not visible on angiography but is detectable on abdominal ultrasound or CT. In such cases, reports of approaches under percutaneous guidance have been documented.^174^, ^175^ Studies on EUS‐guided approaches include cases with previous surgical clipping, where vascular intervention was challenging due to the invisibility of DSA and failed angiographic embolization because of complex splenic artery catheterization difficulties.^91^, ^176^, ^177^ These studies suggest that thrombin injection under EUS guidance could be a new option for pseudoaneurysm management. However, there are unresolved issues regarding endovascular approaches, such as the choice of coils, adhesive agents, or thrombin preparations, in the practice of angioembolization. The 2018 ESGE guidelines recommend arterial embolization before endoscopic drainage for pseudoaneurysms associated with CP; however, this recommendation is supported by “low‐quality evidence.”^91^ Currently, the options for endovascular treatment of CP are unclear. Therefore, accumulating enough cases is crucial to assess the appropriateness of an EUS‐guided approach, even if endovascular treatment is viable.

## FUTURE PERSPECTIVES OF ENDOSCOPIC TREATMENT OF CP

Endoscopic management of CP is a rapidly expanding field. Emerging techniques such as POP‐guided LL and therapeutic EUS show promising efficacy and safety. However, these methods for PDS management are currently off‐label and not guideline‐compliant. Sole reliance on endoscopic therapy may overlook the need for surgical treatment. Further studies are needed for future clinical applications.

## CONFLICT OF INTEREST

Authors declare no conflict of interest for this article.

## FUNDING INFORMATION

None.
